# A Software Platform for Quadruped Robots with Advanced Manipulation Capabilities

**DOI:** 10.3390/s23198247

**Published:** 2023-10-04

**Authors:** Jae-Bong Yi, Shady Nasrat, Min-seong Jo, Seung-Joon Yi

**Affiliations:** Department of Electrical Engineering, Pusan National University, Busan 46241, Republic of Korea; niteofhunter@pusan.ac.kr (J.-B.Y.); shadyloai@pusan.ac.kr (S.N.); jominseong@pusan.ac.kr (M.-s.J.)

**Keywords:** quadruped robot, organize objects, mobile manipulation

## Abstract

Recently, a diverse range of robots with various functionalities have become a part of our daily lives. However, these robots either lack an arm or have less capable arms, mainly used for gestures. Another characteristic of the robots is that they are wheeled-type robots, restricting their operation to even surfaces. Several software platforms proposed in prior research have often focused on quadrupedal robots equipped with manipulators. However, many of these platforms lacked a comprehensive system combining perception, navigation, locomotion, and manipulation. This research introduces a software framework for clearing household objects with a quadrupedal robot. The proposed software framework utilizes the perception of the robot’s environment through sensor inputs and organizes household objects to their designated locations. The proposed framework was verified by experiments within a simulation environment resembling the conditions of the RoboCup@Home 2021-virtual competition involving variations in objects and poses, where outcomes demonstrate promising performance.

## 1. Introduction

Robots have been developed from performing repetitive tasks solely in industrial settings to becoming a part of our daily lives, thanks to advancements in software, sensors, and processors. Notably, recent breakthroughs in machine learning have enabled robots to adeptly perceive their surroundings and engage in natural language communication with humans [1]. Consequently, we now encounter robots operating in diverse environments such as city halls [2], museums [3,4,5], airports [6], and restaurants [7,8,9]. These robots offer interactive and intelligent assistance without relying on specific infrastructures as well as mere repetitive tasks.

However, most robots adopted in ordinary spaces have wheeled locomotion, which presents challenges when encountering obstacles like stairs or thresholds. Moreover, the design of manipulators is often characterized by limited capabilities, primarily encompassing basic gestures and actions.

Lately, studies have been conducted on home service robots designed as mobile manipulators to create practical automated mobile manipulation systems for home environments [1,10,11,12]. However, these investigations only focus on wheeled-type robots equipped with manipulators. Several studies proposed frameworks to conduct grasping tasks with a manipulator mounted on quadruped robot [13,14]. However, many of these frameworks did not include comprehensive tasks combining perception, navigation, locomotion, and manipulation.

In this context, we introduce the software framework to enable a quadruped robot to organize household objects to appropriate space in a domestic environment. Unlike previous works [13,14] that perform just simple mobile manipulation with quadruped robots, our research presents the method for delivering practical services through the utilization of quadruped robots.

**Figure 1:**
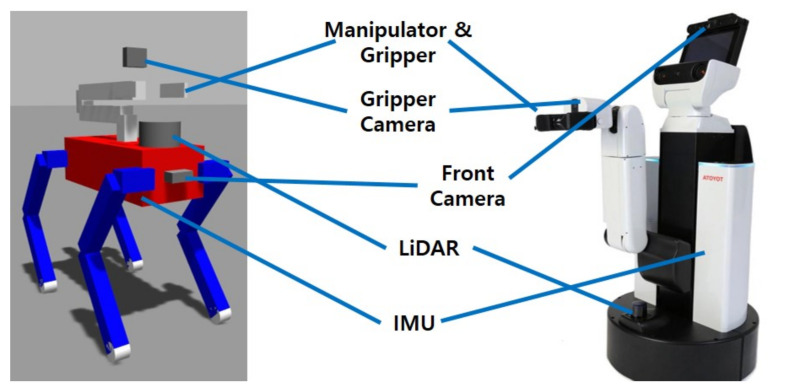
Feature comparison with a robot model for the framework and HSR.

Compared to other platforms that performed similar tasks, a quadruped robot used for this framework should also equipped with cameras, LiDAR, IMU, and a manipulator. Figure 1 shows the feature comparison of the robot model to apply to the framework and to Human Support Robots (HSRs) [15], which performed similar tasks in [1,10,11].

The subsequent sections of this paper are structured as follows: Section 3 describes the overall system of this framework. In Section 4, the method for object detection and point cloud generation is detailed, involving a combination of machine learning techniques including YOLOv7 [16], K-Nearest Neighbor (KNN) [17], and Random Sample Consensus (RANSAC) [18], and the estimation of a grasp pose from a point cloud, accomplished through its conversion into a height map, is presented. Moving to Section 5, navigation strategies outlining how the robot plans its route to the designated area considering the positions of detected objects and LiDAR data are presented, and grasping methods, depending on the situation, are addressed. The mathematical analysis of manipulation and locomotion using Model Predictive Control (MPC) [19,20,21,22] are presented in Section 6. Section 7 showcases the experimental outcomes conducted within a simulation environment resembling RoboCup@Home 2021—virtual. Lastly, the paper concludes by discussing future works in Section 8 and Section 9.

## 2. Related Work

Various service robots deployed in public places were presented in [4,6]. In [4], the “Lindsey” robot, stationed at the Lincoln Museum, successfully operated autonomously as a guide, providing informative tours to visitors. Despite its practical utility, the platform’s lack of physical interaction capabilities limited its scope. A similar case is presented in [6], where the “Spencer” robot facilitated passenger assistance and guidance at Dutch KLM airports. However, this robot also lacks a manipulator for physical engagement. Addressing this limitation, refs. [1,10] introduced a modular software framework for home service robots equipped with manipulators. This comprehensive framework encompassed navigation, visual perception, manipulation, human–robot interaction, and natural language processing. The framework incorporated deep-learning-based perception packages, such as YOLOv3 and OpenPose, to perceive surroundings and combine them with manipulation or navigation tasks using ROS middleware. Depending on its detected data, the framework showed various manipulation skills implemented in robot competitions. This framework showcased promising results in RoboCup@Home 2020 and World Robot Summit 2020 Partner Robot Challenge (WRS 2020 PRC) league using the Toyota HSR [15]. It is worth noting that this system is primarily applicable to wheeled-type mobile manipulators.

Several studies have been conducted regarding quadruped robots equipped with manipulators. In [13], researchers detailed strategies that control a quadruped robot with a Whole Body Control (WBC) framework for arm-mounted quadruped robots. In this work, the author proposed two control modes: manipulation mode and loco-manipulation mode. In the manipulation mode, the author used Hierarchical Quadratic Programming (HQP) [23] to control the arm, legs, and base subject to the whole rigid-body dynamics of the robot. In the loco-manipulation mode, the author controlled the arm with PD control, while the HQP controller controlled the base and the legs. It showed stable gaiting in complex terrains with an arm-mounted quadruped robot. However, this approach did not incorporate image or LiDAR data.

In [14], a comparable system was introduced. The framework was experimented with the quadruped robots equipped with a five Degree of Freedom (DOF) manipulator, front camera, and 3D LiDAR. Using Yolov5, it successfully detected an object to grasp in a 3D position. Additionally, it presented human following with LiDAR data. However, it had limitations in addressing the manipulation of complex-shaped objects like bowls and exclusively concentrated on object manipulation on the ground. Moreover, comprehensive experimental testing of the system’s capabilities was lacking.

In [24], researchers outlined a methodology for grasping complex-shaped objects utilizing an anthropomorphic robotic hand affixed to a seven-DOF arm through imitation learning. By combining 3D reconstruction, KNN, and image recognition using a Residual Neural Network (ResNet), the author realized an imitation learning framework that learns how to grasp complex objects from humans. However, this system required a diverse dataset for learning to grasp, encompassing RGB images, point clouds, and trajectories.

Certain studies have explored methods to grasp detected objects without requiring additional learning [25,26]. In [25], researchers introduced a grasp pose estimation technique based on 3D point cloud analysis, employing Principal Component Analysis (PCA) [27] and RANSAC [18]. While this approach showed promising performance by focusing solely on point cloud contour lines, it was limited in its applicability to objects with complex shapes. Another study, outlined in [26], utilized a virtual gripper with a C-shape configuration to determine the grasp pose. This approach accommodates complex-shaped objects; however, due to the inherently random nature of the deduced grasping orientation, it demands a high-DOF manipulator to secure the object effectively

## 3. System Overview

**Figure 2:**
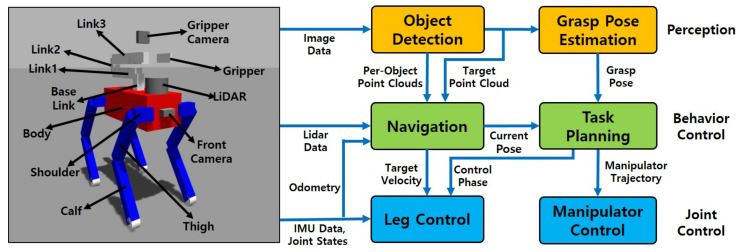
Overview of the system.

Figure 2 shows the simulation model used in this work and the schematics of the framework designed to perform tidy-up tasks that require perception and mobile manipulation with a quadruped robot. To execute the main functions, the model has the form of a quadruped robot equipped with a front camera, gripper camera, LiDAR, and a low-DOF manipulator. The framework is combined with multiple modules interconnected through ROS [28] messages, which are divided into three blocks: perception, behavior control, and joint control. The approximate role of each block is as follows, and Table 1 shows the dimensions of the robot model used in the experiment.

### 3.1. Perception

The perception block is the initial step in our research workflow, encompassing the object detection and grasp pose estimation modules. In the object detection module, we employ a machine learning-based algorithm to process image data, generating point clouds for each detected object. Subsequently, from these point clouds, we select the target point cloud for grasping and derive the grasp pose for the respective object in the grasp pose estimation module.

### 3.2. Behavior Control

The prior information required for joint control is derived in behavior control. By combining LiDAR data and odometry with per-object point cloud and target object, which is derived in the object detection module, the navigation module can generate the target velocity of Center of Mass (COM) and current pose on the map. The current pose and grasp pose are used to derive the control phase, which decides the robot’s control state (e.g., walking or standing) and manipulation trajectory. The task planning module generates the manipulator trajectory when the current grasp pose is appropriate.

### 3.3. Joint Control

The joint control block performs actual roles in moving the robot. The leg control module employs the MPC-based method for precise and stable control. This method requires IMU data, joint states (e.g., position and velocity), and odometry. This module is rooted in [20], and we customize it to suit this research. On the other hand, the manipulator control module utilizes position control using the numerical solution of the inverse kinematics.

## 4. Perception

In order to detect each object in 3D space, we employ a combination of machine learning approaches, including Yolov7, KNN, and RANSAC. Initially, we select the target object from among the detected objects using these methods. Subsequently, we estimate the grasp pose of the chosen object by converting the point cloud into a height map.

### 4.1. Per-Object Point Cloud Generation

**Figure 3:**
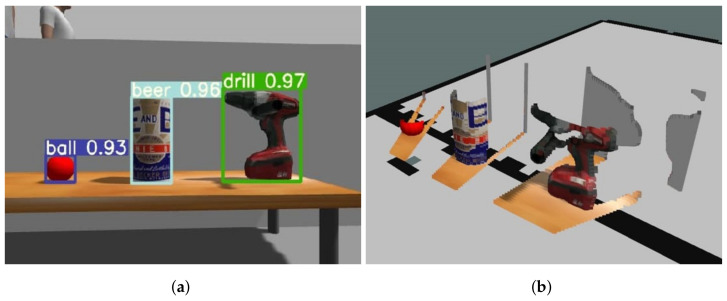
Object detection: (**a**) Detecting objects with YOLOv7 and (**b**) Point clouds per object.

To obtain 3D information about the objects in determining which object to grasp and estimating its grasp pose, generating point clouds for each object emerges as a preliminary step. This endeavor follows the real-time detection of objects from 2D images. As illustrated in Figure 3a, we employ YOLOv7 [16], using Deep Learning methodologies to detect objects and outline their bounding boxes within RGB images in real-time. After object detection, we segment the corresponding positions in the depth image. By projecting this segmented data into 3D space via the intrinsic K matrix [29], point clouds for each object are derived, as shown in Figure 3b.

### 4.2. Filtering Outliers

**Figure 4:**
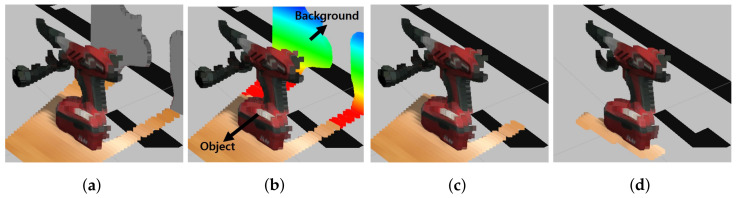
Process of filtering outliers: (**a**) Original, (**b**) Part division, (**c**) Background removal, and (**d**) Filtering outliers.

Figure 4a reveals the presence of outliers causing distortions. To address this issue, we deploy two machine learning techniques: KNN [17] and RANSAC [18]. Initially, showcased in Figure 4b, we partition the point cloud into object and background segments using KNN [17]. However, distinguishing between the object and background through KNN [17] alone is challenging. To address this, considering the closer proximity of the object’s centroid to the robot’s camera, we exclude the background by eliminating the portion with a more distant centroid. This strategy yields a model devoid of background, showcased in Figure 4c. Subsequently, by leveraging plane-fitting RANSAC [18], the remaining outliers are filtered, aiding in the acquisition of plane normal vectors utilized for predicting grasp directions. The culmination of these steps yields refined 3D models free from outliers, as depicted in Figure 4d.

### 4.3. Probing Direction Decision

**Figure 5:**
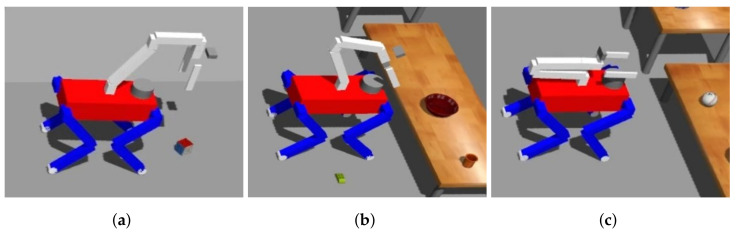
Setting probing posture in probing area: (**a**) Vertical-floor, (**b**) Vertical-table, and (**c**) Horizontal.

Depending on the object’s state, such as on the floor or the table, and properties, the robot should determine its probing direction to estimate a grasp pose of the object in the easy-to-grasp posture. The robot employs a gripper-mounted camera to probe the object vertically when the object is positioned on the floor, as exemplified in Figure 5a. However, for objects on the table, the probing direction requires prediction. This prediction entails adopting the posture depicted in Figure 5b for object assessment. Within this configuration, the robot employs the plane-fitting RANSAC [18] to compute the normal vector of the object’s point cloud. If the *z*-coordinate of this normal vector surpasses a predetermined threshold, the robot concludes that vertical probing is ideal and proceeds to generate a corresponding height map. Conversely, horizontal probing is deemed more suitable if the *z*-coordinate falls below the threshold. In this case, the robot repositions its manipulator to the configuration shown in Figure 5c and adjusts its position to a grasp-appropriate point. Subsequently, with the manipulator reoriented, the robot employs its body-mounted front camera to create a height map for the object’s horizontal probing assessment.

### 4.4. Height Map Creation

**Figure 6:**
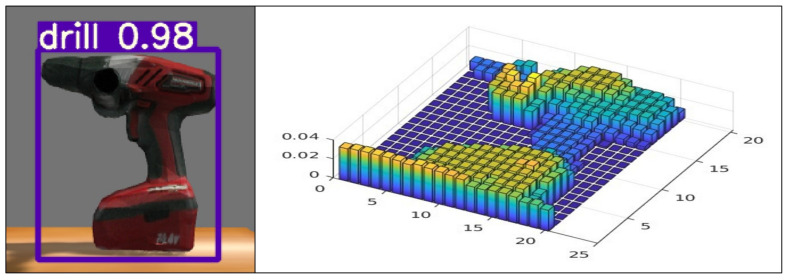
Height map.

The construction of a height map, derived from the point cloud data, is executed through distinct coordinate configurations according to the robot’s chosen probing direction. In instances where the robot decides on horizontal probing, the *x* and *y* coordinates of the height map are extracted from the point cloud’s *y* and *z* coordinates, respectively. Conversely, for vertical probing, the *x* and *y* coordinates of the height map are derived from the point cloud’s *x* and *y* coordinates, correspondingly. The height values assigned to the height map are drawn from the *z*-coordinates of the point cloud when probing vertically, while horizontal probing utilizes the *x*-coordinates for height value determination. A detailed explanation is given in Algorithm 1, and Figure 6 shows the result.
**Algorithm 1** Height map creation
1:
x_coords ← removeDuplicate(target_point_cloud.x)
2:
y_coords ← removeDuplicate(target_point_cloud.y)
3:
z_coords ← removeDuplicate(target_point_cloud.z)
4:
y_unit ← y_coords.size / HEIGHT_MAP_SIZE
5:**if** probing_pose is horizontal **then**6:    
x_unit ← z_coords.size / HEIGHT_MAP_SIZE
7:    
creteria_height ← max(target_point_cloud.x)
8:**else**9:    
x_unit ← x_coords.size / HEIGHT_MAP_SIZE
10:   
creteria_height ← min(target_point_cloud.z)
11:**end if**12:**for** i←1 to target_point_cloud.size **do**13:    
height_map_x ← rank(target_point_cloud.y[i],y_coords)
14:    **if** probing_pose is horizontal **then**15:        
point_height ← creteria_height−target_point_cloud.x[i]
16:        
height_map_y ← rank(target_point_cloud.z[i],z_coords)
17:    **else**18:        
point_height ← target_point_cloud.z[i]−creteria_height
19:        
height_map_y ← rank(target_point_cloud.x[i],x_coords)
20:    **end if**21:    **for** j←1 to HEIGHT_MAP_SIZE **do**22:        **if** height_map_x≥x_unit∗(j−1) and height_map_x≤x_unit∗j **then**23:           **for** k←1 to HEIGHT_MAP_SIZE **do**24:               **if** height_map_y≥y_unit∗(k−1) and height_map_y≤y_unit∗k **then**25:                     
height_map_num(j,k) ← height_map_num(j,k)+1
26:                     
height_map_sum(j,k) ← height_map_sum(j,k)+point_height
27:               **end if**28:           **end for**29:        **end if**30:    **end for**31:**end for**32:
height_map ← height_map_sum / height_map_num



### 4.5. Grasp Pose Prediction

The height map derived in Section 4.4 is used to predict grasp pose. From this map, we select grasp candidates and select grasp pose among them, considering contact pose inclination and distance to the center of the height map. Subsequently, we convert the grasp pose, initially represented in the height map, into the 3D space.

#### 4.5.1. Selecting Grasp Candidates

**Figure 7:**
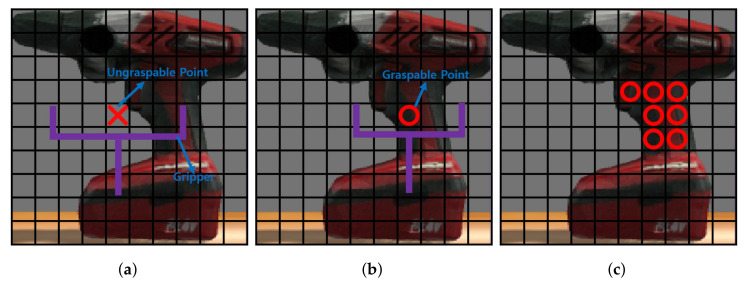
Selecting grasp candidates: (**a**) Ungraspable position, (**b**) Graspable position, and (**c**) Grasp candidates.

The primary step in the prediction process involves the selection of grasp candidates extracted from the height map. As illustrated in Figure 7, this procedure requires transforming the gripper to fit the height map and subsequently evaluating each element in conjunction with the gripper configuration to ascertain its graspability. A point is considered a graspable candidate when the height of the coordinate situated at the center of the gripper exceeds the height of the locations where the gripper’s tips are positioned by a predefined margin. However, within this evaluation, if the slopes present within the gripper’s region exhibit a gradient lower than a specified threshold, the coordinate is classified as ungraspable. Comprehensive details of this operational phase are presented in Algorithm 2.
**Algorithm 2** Selecting grasp candidates
1:**for** i←1 to HEIGHT_MAP_SIZE **do**2:    **for** j←1 to HEIGHT_MAP_SIZE **do**3:        
left_tip_pos ← i+gripper_width_half_height_map
4:        
right_tip_pos ← i−gripper_width_half_height_map
5:        
left_tip_diff ← height_map(i,j)−height_map(left_tip_pos,j)
6:        
right_tip_diff ← height_map(i,j)−height_map(right_tip_pos,j)
7:        **if** left_tip_diff or right_tip_diff > GRASPABLE_HEIGHT **then**8:           
grasp_pos_found ← false
9:           **for** k←right_tip_pos to i−1 **do**10:               
right_height_slope ← height_map(k+1,j)−height_map(k,j)
11:               **if** right_height_slope≥GRASPABLE_HEIGHT_VAR **then**12:                   **for** l←i+1 to left_tip_pos **do**13:                       
left_height_slope ← height_map(l−1,j)−height_map(l,j)
14:                       **if** right_height_slope≥GRASPABLE_HEIGHT_VAR **then**15:                          
grasp_pos_candidates.add({i,j})
16:                          
right_tip_contact_poses.add({k,j})
17:                          
left_tip_contact_poses.add({l,j})
18:                          
grasp_pos_found ← true
19:                          
break
20:                       **end if**21:                   **end for**22:               **end if**23:               **if** grasp_pos_found **then**24:                   
break
25:               **end if**26:           **end for**27:        **end if**28:    **end for**29:**end for**


#### 4.5.2. Getting Contact Pose Inclination

While candidates might meet the criteria outlined in Section 4.5.1, addressing potential slipping issues arising from unaccounted contact pose inclinations is essential. To address this concern, we engage neighboring contact coordinates around the present contact coordinate to ascertain inclinations. This involves determining slopes based on contact coordinates adjacent to the existing contact coordinate, thus enabling the derivation of contact pose inclinations. Algorithm 3 details the precise steps.
**Algorithm 3** Getting contact pose inclination
1:**for** i←1 to HEIGHT_MAP_SIZE **do**2:    **for** j←1 to HEIGHT_MAP_SIZE **do**3:        
vicinity_pose_x.empty()
4:        
vicinity_pose_y.empty()
5:        **if** tip_contacted_pos.is_exist({i,j}) is true **then**6:           **for** k←−VICINE_RANGE to VICINITY_RANGE **do**7:               **for** l←−VICINE_RANGE to VICINITY_RANGE **do**8:                   **if** tip_contacted_pos.is_exist({i+k,j+l}) is true **then**9:                       
vicinity_pose_x.add(i+k)
10:                      
vicinity_pose_y.add(j+l)
11:                   **end if**12:               **end for**13:           **end for**14:           
vicinity_pose_x_diff ← vicinity_pose_x.max−vicinity_pose_x.min
15:           
vicinity_pose_y_diff ← vicinity_pose_y.max−vicinity_pose_y.min
16:           
tip_pose_inclination.add(atan2(vicinity_pose_y_diff,vicinity_pose_x_diff))
17:        **end if**18:    **end for**19:**end for**


#### 4.5.3. Selecting Grasp Pose in Height Map

**Figure 8:**
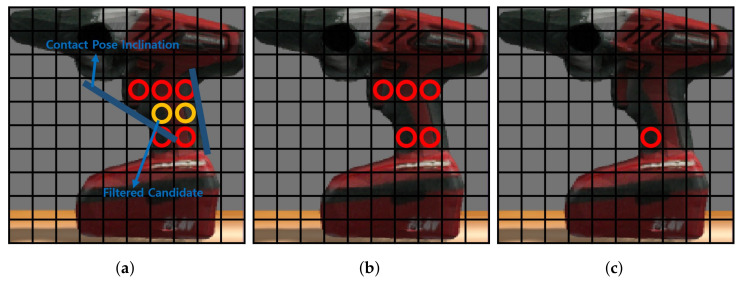
Selecting grasp pose: (**a**) Getting contact inclination, (**b**) Filtering candidates, and (**c**) Selecting grasp pose.

Following determining the contact pose inclinations, depicted in Figure 8, a subsequent filtering process is implemented to address candidates within low inclination regions. From the remaining candidates, the one closest to the center of the height map is selected as the prime candidate. In cases where multiple candidates share the same distance to the center, the selection prioritizes the candidate within the narrowest area.

#### 4.5.4. Grasp Pose Transition

Concluding the prediction process, the final step is translating the grasp pose determined within the height map to a comprehensive 3D pose, accomplished through Algorithm 4. This process effectively reverses the steps undertaken in Algorithm 1, utilizing derived variables such as x_coords , y_coords , and z_coords from the earlier algorithm.

**Figure 9:**
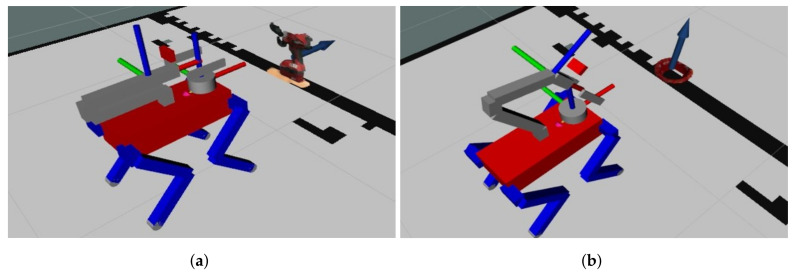
Grasp pose in 3D point cloud: (**a**) Drill and (**b**) Bowl.

The outcome of Algorithm 4 is showcased in Figure 9, where the position of the arrow symbolizes the grasp pose. At the same time, its orientation represents the derived grasp direction, facilitated by utilizing the normal vector from the plane-fitting RANSAC [18]. This step finalizes the prediction procedure, ensuring accurate grasp pose representation in three-dimensional space.
**Algorithm 4** Grasp pose transition
1:**if** probing_pose is horizontal **then**2:    
grasp_pos_3d.x←creteria_height−height_map(grasp_pos_2d.x,grasp_pos_2d.y)
3:**else**4:    
grasp_pos_3d.z←height_map(grasp_pos_2d.x,grasp_pos_2d.y)+creteria_height
5:**end if**6:

grasp_pos_idx_horizontal←grasp_pos_2d.y∗y_unit
7:

grasp_pos_idx_vertical←grasp_pos_2d.x∗x_unit
8:

grasp_pos_3d.y←y_coords[grasp_pos_idx_horizontal]
9:**if** probing_pose is horizontal **then**10:    
grasp_pos_3d.z←z_coords[grasp_pos_idx_vertical]
11:**else**12:    
grasp_pos_3d.x←x_coords[grasp_pos_idx_vertical]
13:**end if**


### 4.6. Rotation of an Object on The Floor

**Figure 10:**
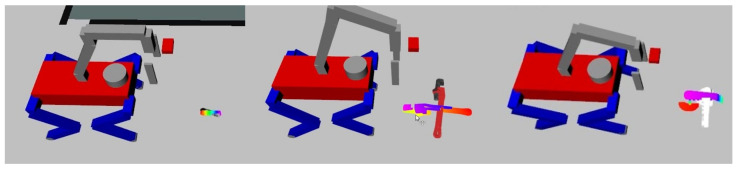
Rotation of an object on the floor.

When dealing with objects situated on the floor, achieving an optimal grasp is facilitated when the object’s orientation aligns with the gripper’s wrist angle. To achieve this alignment, we leverage the line-fitting RANSAC [18] when probing objects on the floor. As illustrated in Figure 10, the direction of the object’s normal vector corresponds to the desired gripper angle. To accommodate this alignment, we perform a *z*-axis rotation of the object’s point cloud according to the normal vector, preceding the grasp pose prediction step. The final execution involves the robot grasping the object using the gripper positioned in alignment with the object’s orientation, thus optimizing the grasping process for objects located on the floor.

## 5. Behavior Control

Before engaging in robot control at the joint level, managing and directing the robot’s behavior is essential. Based on the detection information discussed in Section 4, the robot performs Navigation and decides grasping form.

### 5.1. SLAM

**Figure 11:**
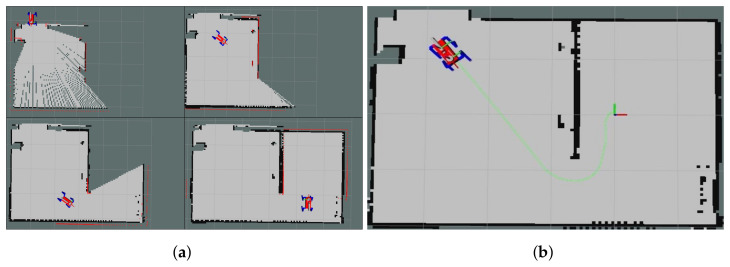
SLAM and navigation: (**a**) SLAM and (**b**) Navigation.

In preparation for organizing objects, the robot initiates its process by determining its position and comprehending its immediate environment. This initial phase involves the creation of a spatial map using ROS’s SLAM package known as gmapping.Through manual guidance within the designated area, the robot creates a map using LiDAR data and odometry, as illustrated in Figure 11a. As the locomotion algorithm used in this framework generates less staggering in gaiting, additional compensations are not required.

### 5.2. Navigation

For precise navigation to predetermined positions within the map established in Section 5.1, the robot’s movement is facilitated by utilizing the ROS package associated with navigation, known as amcl. While this package has proven effective, it is employed primarily for transporting the robot to designated search or deposit zones due to limitations in accurately approaching goal poses. Figure 11b exemplifies the process involving this package.

### 5.3. Approaching

**Figure 12:**
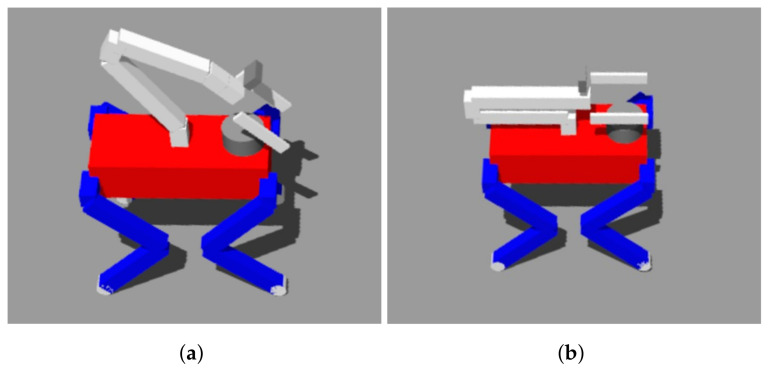
Searching postures: (**a**) Searching floor object posture and (**b**) Searching table object posture.

The robot’s initial task involves identifying graspable objects. By employing a camera attached to the gripper, the robot scans the floor while maintaining the posture displayed in Figure 12a. Upon detecting object centroids within its body frame of reference, the robot adjusts its movement toward the nearest object. Yet, when the proximity to this object falls below a defined threshold, TARGETING_DIST , it is categorized as a target_object . Subsequently, the robot repositions itself to a probing_area , which ensures accessibility by the gripper, as illustrated in Figure 5. Conversely, the robot reconfigures its manipulator to resemble the stance depicted in Figure 12b in scenarios where no floor objects are detected. This alternative posture is employed for surveying objects on a table using the front camera, following a procedure analogous to that used for the floor. Algorithm 5 offers a comprehensive breakdown of this operational phase.
**Algorithm 5** Approaching
1:

searching_mode.manipulator←floor_searching
2:

searching_mode.camera←gripper_camera
3:**if** isObjectExist(searching_state) is false **then**4:    
searching_mode.manipulator←table_searching
5:    
searching_mode.camera←front_camera
6:    
probing_area_type←FLOOR_PROBING_AREA
7:**else**8:    
probing_area_type←TABLE_PROBING_AREA
9:**end if**10:**while** target_object.centroid not in probing_area **do**11:    
object_clouds←searchObjects(searching_state)
12:    **if** target_object_id is null **then**13:        
closest_object←getClosestObject(object_clouds)
14:        
traceObject(closest_object.centroid)
15:        **if** closest_object.dist≤TARGETING_DIST **then**16:           
target_object_id←closest_object.object_id
17:        **end if**18:    **else**19:        
target_object←getTragetObject(object_clouds,target_object_id)
20:        
moveToProbingArea(target_object.centroid,probing_area_type)
21:    **end if**22:**end while**


### 5.4. Grasping an Object

**Figure 13:**
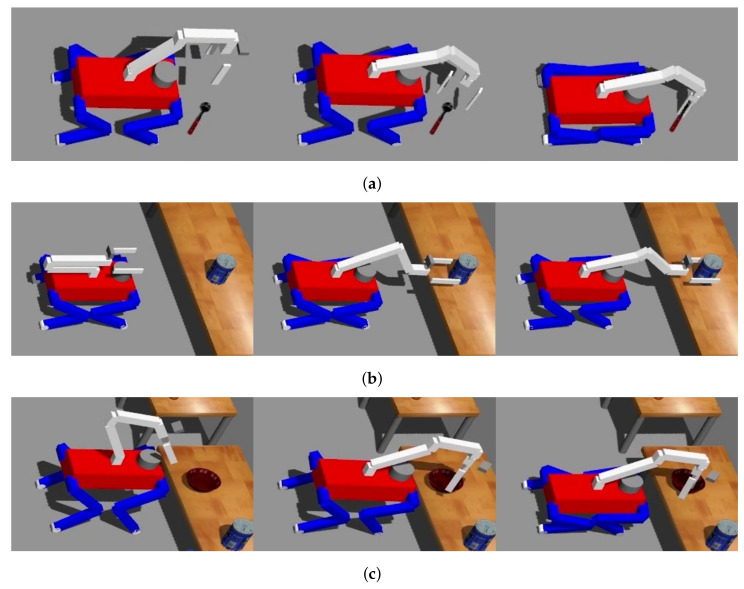
Grasping an object differently depending on the situation: (**a**) Grasping an object on the floor, (**b**) Grasping an object on the table horizontally, and (**c**) Grasping an object on the table vertically.

The act of grasping is executed with variations contingent upon the object’s specific situation, as demonstrated in Figure 13. Despite these variations, the fundamental grasping process can be categorized into three distinct stages: probing, transitioning to a grasp-ready pose, and actual grasping.

Upon reaching an area conducive to grasping, the robot initiates the probing stage, which adapts according to the specific scenario. After probing, the robot adjusts the positioning of its COM and manipulator to align the grasp pose within the object with the gripper’s front. In the ensuing stage, when the grasp direction is vertical, the robot adjusts its COM along the *z*-axis by flexing its knee, effectively facilitating object grasping. Conversely, for horizontal grasp directions, the robot shifts its manipulator along the *x*-axis to the object’s location, preventing any potential collision between the gripper and the object. However, when the object’s distance exceeds the manipulator’s operational range, the robot compensates by moving its COM along the *x*-axis. Algorithm 6 comprehensively describes this process.

**Algorithm 6** Grasping an object
1:**if** grasp_ready_phase **then**2:    
moveCOM(−grasp_pos_3d.y)
3:    **if** grasp_orientation is vertical **then**4:        
gripper_pose.x←grasp_pose_3d.x
5:        
gripper_pose.z←grasp_pose_3d.z+GRASP_READY_Z
6:        **if** centoid_grasp_pose_diff_dist≥ROLL_THRESHOLD **then**7:           
gripper_roll←atan2(centoid_grasp_pose_diff.y,centoid_grasp_pose_diff.x)
8:        **else**9:           
gripper_roll←line_fitting_RANSAC(filtered_point_cloud).normal
10:       **end if**11:    **else**12:        
gripper_pose.x←GRASP_READY_X
13:        
gripper_pose.z←grasp_pose_3d.z
14:    **end if**15:**else if** grasp_phase **then**16:    **if** grasp_orientation is vertical **then**17:          
grapper_pos.z←grasp_pos_3d.z+COM_MOVE_Z
18:          
com_pose.z−=COM_MOVE_Z
19:    **else**20:        **if** grasp_pos_3d.x≤MANIPULATOR_X_LIM **then**21:             
grapper_pos.x←grasp_pos_3d.x
22:        **else**23:             
grapper_pos.x←MANIPULATOR_X_LIM
24:             
com_pose.x+=(grasp_pos_3d.x−MANIPULATOR_X_LIM)
25:        **end if**26:    **end if**27:**end if**


## 6. Joint Control

To facilitate the execution of desired robot behaviors, control at the joint level should be performed. The robot’s motion control comprises two essential components: Manipulator Control and Leg Control. The robot controls its manipulator with position control with a numerical solution of inverse kinematics and controls its leg with the MPC-based method. Table 2 shows the dimensions of the manipulator.

### 6.1. Manipulator Control

**Figure 14:**
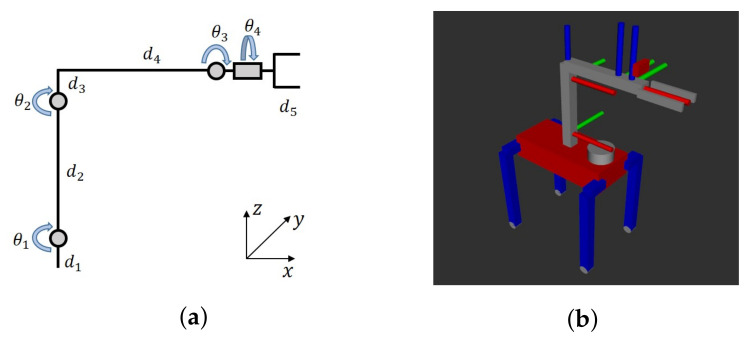
Manipulator configuration and model: (**a**) Arm configuration and (**b**) Robot model.

To ensure minimal impact on the robot’s gaiting, the manipulator integrated onto the quadruped robot is designed to be lightweight. Achieving this objective involves employing a low-DOF manipulator, effectively reducing the weight of its actuators. As depicted in Figure 14, a four-DOF manipulator configuration has been adopted for object grasping. Utilizing these parameters, we can define $T_{tr}\left(x,y,z\right)$ as a translational transform and $R_{x}$ , $R_{y}$ , and $R_{z}$ as rotational transforms around the *x*, *y*, and *z* axes, respectively. Consequently, the solution for the arm’s forward kinematics can be deduced as follows:

(1) $$T_{\mathrm{manipulator}}=T_{tr}\left(0,0,d_{1}\right)R_{y}\left(\theta_{1}\right)T_{tr}\left(0,0,d_{2}\right)R_{y}\left(\theta_{2}\right)T_{tr}\left(d_{4},0,d_{3}\right)R_{y}\left(\theta_{3}\right)R_{x}\left(\theta_{4}\right)T_{tr}\left(d_{5},0,0\right)$$

#### Inverse Kinematics

To obtain the inverse kinematics solution for a target transform $T_{\mathrm{manipulator}}$ , we first calculate the relative wrist position $\left(x^{\prime },0,z^{\prime }\right)$ .

(2) $${T\prime }_{\mathrm{manipulator}}=T_{tr}\left(0,0,-d_{1}\right)T_{\mathrm{manipulator}}T_{tr}\left(-d_{5},0,0\right)$$

(3) $${\left[\begin{array}{cccc} x^{\prime } & 0 & z^{\prime } & 1 \end{array}\right]}^{T}={T\prime }_{\mathrm{manipulator}}{\left[\begin{array}{cccc} 0 & 0 & 0 & 1 \end{array}\right]}^{T}$$

**Figure 15:**
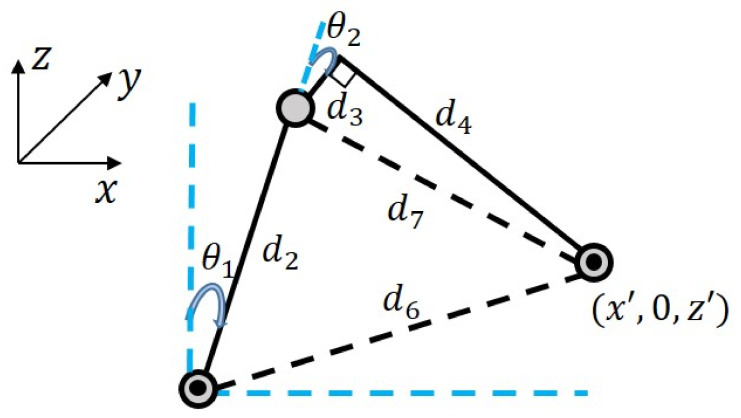
Arm configuration without the gripper and base.

Using the components in Figure 15, we can obtain $\theta_{1}$ and $\theta_{2}$ with following equation.

(4) $$d_{6}=\sqrt{{x\prime }^{2}+{z\prime }^{2}}$$

(5) $$d_{7}=\sqrt{d_{3}^{2}+d_{4}^{2}}$$

(6) $$\theta_{1}=\frac{\pi }{2}-a\cos\left(\frac{d_{1}^{2}+d_{6}^{2}-d_{7}^{2}}{2d_{1}d_{6}}\right)-a\tan2\left(z^{\prime },x^{\prime }\right)$$

(7) $$\theta_{2}=\pi -a\cos\left(\frac{d_{1}^{2}+d_{7}^{2}-d_{6}^{2}}{2d_{1}d_{7}}\right)-a\tan2\left(d_{4},d_{3}\right)$$

As all joints without $\theta_{4}$ are moved in the *y*-axis, the gripper’s roll is the same with $\theta_{4}$ . Now that we know $\theta_{1}$ , $\theta_{2}$ , and $\theta_{4}$ , we can obtain $\theta_{3}$ with following equation:

(8) $$R_{y}\left(\theta_{3}\right)=T_{tr}\left(-d_{4},0,-d_{3}\right)R_{y}\left(-\theta_{2}\right)T_{tr}\left(0,0,-d_{2}\right)R_{y}\left(-\theta_{1}\right){T\prime }_{\mathrm{manipulator}}R_{x}\left(-\theta_{4}\right)$$

(9) $$\theta_{3}=a\cos\left(R_{y}{\left(\theta_{3}\right)}_{11}\right)$$

### 6.2. Leg Control

In Leg Control, we adopted a framework rooted in MPC, introduced in [20]. Considering its current state and desired pose, this framework derives the appropriate Ground Reaction Force (GRF) with MPC. To adopt this framework as the Leg Control module, we adjusted several components to fit this work.

#### 6.2.1. COM Controller

As the Navigation module returns the target velocity, we adjust the desired pose with this value. When the target velocity is returned, the desired pose is adjusted as follows.

(10) $$p_{target}\left(t+\Delta t\right)=p_{target}\left(t\right)+v_{target}\Delta t$$

(11) $$\Omega_{target}=\left[\begin{array}{cccc} 0 & -\omega_{target\_x} & -\omega_{target\_y} & -\omega_{target\_z}\\ \omega_{target\_x} & 0 & \omega_{target\_z} & -\omega_{target\_z}\\ \omega_{target\_y} & -\omega_{target\_z} & 0 & \omega_{target\_x}\\ \omega_{target\_z} & \omega_{target\_y} & -\omega_{target\_x} & 0 \end{array}\right]$$

(12) $$q_{target}\left(t+\Delta t\right)=\left(I+\frac{1}{2}\Omega_{target}\Delta t\right)q_{target}\left(t\right)$$

Subsequently, we set VEL_LIMIT in FSM the same as an absolute value of the target velocity.

#### 6.2.2. Gait

**Figure 16:**
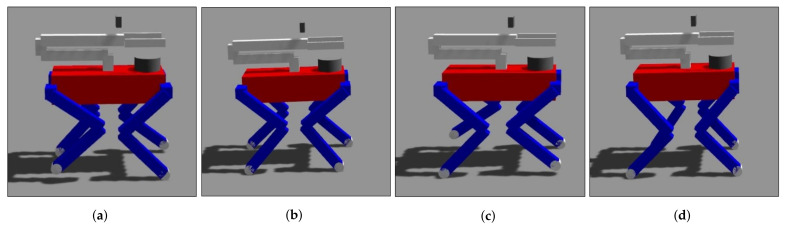
Gaiting with four phases: (**a**) swing_FLRR, (**b**) stance_FLRR, (**c**) swing_FRRL, and (**d**) stance_FRRL.

In navigation, we adopt trotting as a gaiting form. As shown in Figure 16, the trotting phase is divided into four phases: swing_FLRR, stance_FLRR, swing_FRRL, and stance_FRRL. The desired GRF in swing phases (swing_FLRR and swing_FRRL) is half of the body mass, and the desired GRF in stance phases (stance_FLRR and stance_FRRL) is a quarter of the body mass. Since the leg mass is less than 10% of the robot’s total mass, the legs’ inertia effect could be neglected.

#### 6.2.3. Parameters

The parameters used in Leg Control should be adjusted to fit the environment of this work. Table 3 shows the parameters used in this work. In this table, $R_{u}$ , Q , $N_{hor}$ , $T_{sw}$ , $T_{st}$ , and $T_{pred}$ represent input weight matrix, pose weight matrix, expectation horizon, swing foot time, stance foot time, and prediction time, respectively.

#### 6.2.4. Finite State Machine

This segment defines the robot’s reference state utilized in the MPC framework. Under normal circumstances, we presume that the robot’s initial posture and velocity mirror the current state, aiming to refine its velocity by controlling acceleration to achieve the intended pose. Nevertheless, when the current velocity’s trajectory diverges from the desired pose, we adapt the planned velocity to align with the desired pose’s direction, bypassing the current state’s influence. Algorithm 7 provides an intricate breakdown of this process, incorporating parameters such as ADJUST_VEL_THRESHOLD , FEED_BACK_VEL , and PLANNED_ACC , all of which are denoted as positive numerical values.
**Algorithm 7** Finite state machine
1:

now_planned_pose←current_pose
2:

now_planned_vel←current_vel
3:**for** i←1 to EXPECTATION_HORIZON **do**4:    
pose_diff←desired_pose−now_planned_pose
5:    **if** pose_diff.abs<ADJUST_VEL_THRESHOLD **then**6:          
now_planned_vel←0
7:          
now_planned_pose←desired_pose
8:    **else**9:          **if** pose_diff∗now_planned_vel<0 **then**10:           **if** now_planned_vel<0 **then**11:               
now_planned_vel←FEED_BACK_VEL
12:           **else**13:               
now_planned_vel←−FEED_BACK_VEL
14:           **end if**15:        **else**16:           **if** $\frac{now\_planned\_vel^{2}}{2ACCEL}<pose\_diff.abs$ **then**17:               
$now\_planned\_vel-=PLANNED\_ACC*T_{pred}*pose\_diff.sign$
18:           **else if** now_planned_vel.abs≤VEL_LIMIT **then**19:               
$now\_planned\_vel+=PLANNED\_ACC*T_{pred}*pose\_diff.sign$
20:           **end if**21:        **end if**22:        
$now\_planned\_pose+=now\_planned\_vel*T_{pred}$
23:    **end if**24:    
planned_vel[i]←now_planned_vel
25:    
planned_pose[i]←now_planned_pose
26:**end for**


#### 6.2.5. Swing Foot Trajectory

Given the dynamic nature of the desired foot placement position, varying based on the specific leg position (front, rear, left, or right), we employ Algorithm 8 to establish the swing foot trajectory.

**Algorithm 8** Swing foot trajectory
1:

$TIME\_CONST\leftarrow \frac{T_{sw}}{2}+EXTRA\_DIST$
2:**if** is_front **then**3:    
desired_swing_foot_pos.x←BODY_LENGTH_HALF
4:    
$foot\_pos\_des.x\leftarrow (now\_lin\_vel.x-HIP\_JOINT\_Y*\omega_{z})*TIME\_CONST$
5:    
$foot\_pos\_des.y\leftarrow (now\_lin\_vel.y+SHOULDER\_LENGTH*\omega_{z})*TIME\_CONST$
6:**else**7:    
desired_swing_foot_pos.x←−BODY_LENGTH_HALF
8:    
$foot\_pos\_des.x\leftarrow (now\_lin\_vel.x+HIP\_JOINT\_Y*\omega_{z})*TIME\_CONST$
9:    
$foot\_pos\_des.y\leftarrow (now\_lin\_vel.y-SHOULDER\_LENGTH*\omega_{z})*TIME\_CONST$
10:**end if**11:**if** is_left **then**12:    
desired_swing_foot_pos.y←HIP_JOINT_Y
13:**else**14:    
desired_swing_foot_pos.y←−HIP_JOINT_Y
15:**end if**16:

$xy\_pos\_traj\_elem\leftarrow 0.5(1-cos\left(\frac{\pi }{T_{sw}}t\right))$
17:

desired_swing_foot_pos.x+=foot_pos_des.x∗xy_pos_traj_elem
18:

desired_swing_foot_pos.y+=foot_pos_des.y∗xy_pos_traj_elem
19:

$desired\_swing\_foot\_pos.z=TROT\_FOOT\_HEIGHT*sin(\frac{\pi }{T_{sw}}t)$



## 7. Experiment

**Figure 17:**
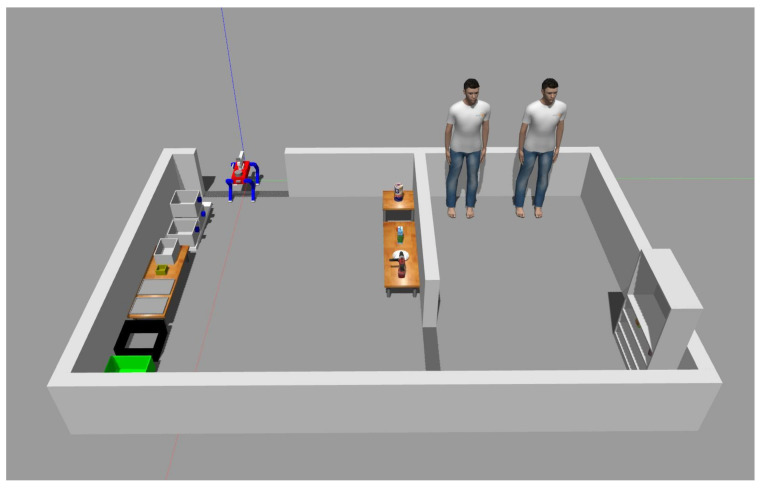
Simulation model and environment.

**Figure 18:**
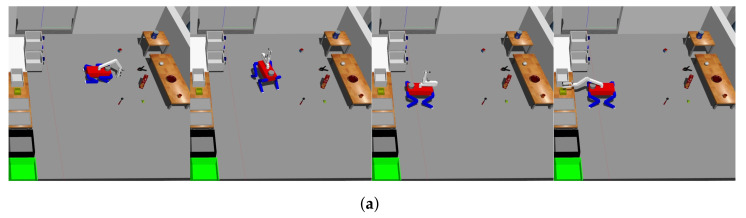

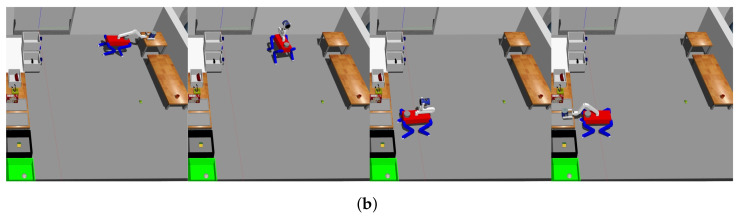
Object classification tasks: (**a**) Classifying an object on the floor and (**b**) Classifying an object on the table.

The experimental evaluation was conducted using Gazebo [30], an open-source 3D robotic simulator integrated within the ROS framework [28]. However, we found a slipping problem when grasping an object with the gripper, so we adopted gazebo_grasp_plugin to solve it. The experimental setup, as illustrated in Figure 17, closely emulated the configuration resembling the Robocup@Home 2021-virtual league environment. As is customary in the competition, shown in Figure 18, this experiment encompassed object classification tasks. The system’s speed and accuracy were assessed by placing objects in predefined positions. To run the simulation and YOLOv7 simultaneously, we used the desktop equipped with an AMD Ryzen 7 5800X, 32 GB of RAM, and an NVIDIA GeForce RTX 3090.

### 7.1. Objects

**Figure 19:**
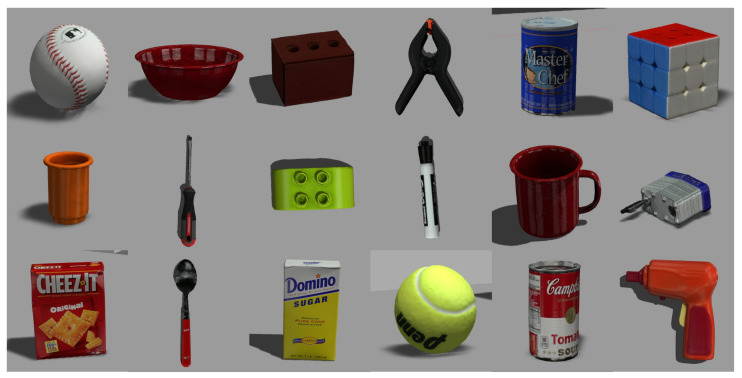
Objects used in the experiment.

A set of eighteen distinct objects was utilized during the experimental phase, as illustrated in Figure 19. These objects include a baseball, bowl, brick, clamp, coffee can, Rubik’s cube, cup, driver, Lego, marker, padlock, cracker, spoon, sugar, tennis ball, tomato soup, and toy gun. These objects are drawn from the YCB object dataset, an official selection used in the Robocup@Home. Throughout the experiment, the objects’ configurations were modified within the environment.

### 7.2. Tasks

**Figure 20:**
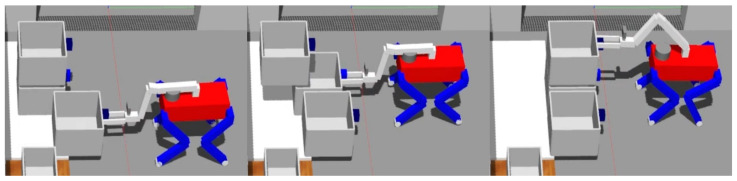
Opening drawer.

The experiment was structured around two main tasks: opening drawers and classifying objects. As depicted in Figure 20, the drawers were positioned in three distinct configurations: left, right, and top. At the outset of the experiment, the initial step involved manipulating the manipulator to open these drawers. Once the drawer-opening task was completed, the robot proceeded to the object classification phase.

**Figure 21:**
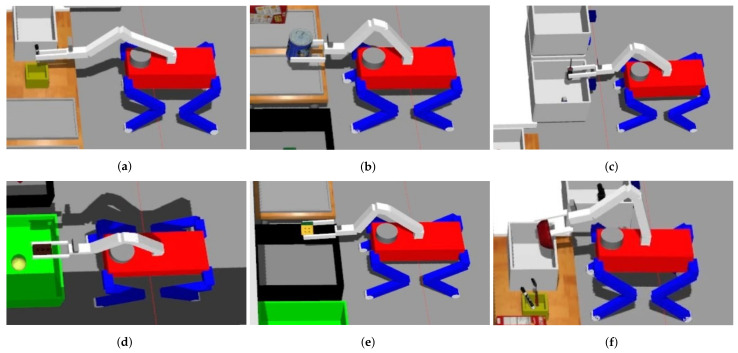
Object classification sections: (**a**) Pencil case, (**b**) Tray, (**c**) Drawer, (**d**) Green box, (**e**) Black box, and (**f**) Small box.

Object classification was executed through the deposition of objects into designated areas corresponding to specific categories. These categories encompassed orientation-based items (e.g., marker, spoon), food items (e.g., coffee can, sugar, tomato soup, and cracker), tools (e.g., driver, clamp, and padlock), shape items (e.g., baseball, tennis ball, cup, and brick), task items (e.g., Rubik’s cube, toy gun, and Lego), and kitchen items (e.g., bowl, mug). The classification process was facilitated by arranging objects in the appropriate area corresponding to their respective categories. This classification was performed within an area divided into six distinct sections, as illustrated in Figure 21: pencil case (orientation-based items), tray (foods), drawer (tools), green box (shape items), black box (task items), and small box (kitchen items).

For a comprehensive visual representation of the experiments, all corresponding video recordings can be accessed at the link https://www.youtube.com/playlist?list=PLB1pUAsYGpRGpUhJ0qVN3_Y0EIwi5TMcz, (accessed on 1 October 2023).

### 7.3. Results

The experiment was repeated across five distinct environments, each involving ten objects from the selection shown in Figure 19. The outcomes of these trials are summarized in Table 4, providing details such as the number of successful attempts, the number of failures, the success rate, and the duration taken for each test. However, as the opening of the drawers succeeded in all experiments, it is not described in the table. Notably, the average success rate across all trials amounted to 96%. Moreover, the success rates for all environments consistently exceeded 80%, underscoring the system’s robust performance across varying contexts. Comparatively, our system exhibited longer task execution times in certain scenarios, such as turning in place, walking sideways, and setting a grasp-ready pose, when compared to a wheeled robot. In particular, our platform required more than 3 min to complete the tasks with six fewer objects than [10], which utilized a wheeled robot (HSR) for a similar experiment.

## 8. Discussion

In this work, we proposed a mobile manipulation framework to organize household objects with quadruped robots. As described in Section 7, the model used in the experiment shows high stability and accuracy in locomotion and manipulation. Additionally, when estimating an object’s grasp pose by combining machine learning algorithms, it selected an appropriate grasp pose in real time for the objects used in the experiment, even for challenging objects such as bowls, toy guns, and cups. However, when comparing its spending time with previous experiments using wheeled robots [10], the system’s operational speed is relatively time-consuming.

Although this work successively reaches the goal of developing a framework to organize household objects with a quadruped robot, it is essential to acknowledge that these accomplishments were obtained exclusively within a simulation environment without tasks executed on uneven terrains, a significant advantage inherent to quadruped robots. Furthermore, the experiment required only mobile manipulation skills without Human–Robot Interaction (HRI) required for the robots used in ordinary places.

To address these limitations, in the following works, we plan to conduct experiments in real-world environments using physical hardware, including scenarios with uneven terrains. Simultaneously, we will committed to optimizing the framework to reduce task completion times and expanding its capabilities to include HRI functionalities.

## 9. Conclusions

This study introduces a comprehensive robotic framework that effectively performs household tasks through the integration of a quadruped robot equipped with perception, navigation, manipulation, and body control. The system’s reliability is underscored by a successful experiment that attests to its high accuracy. However, this research was confined to simulation-based experiments, and task execution times were relatively extended.

**Figure 22:**
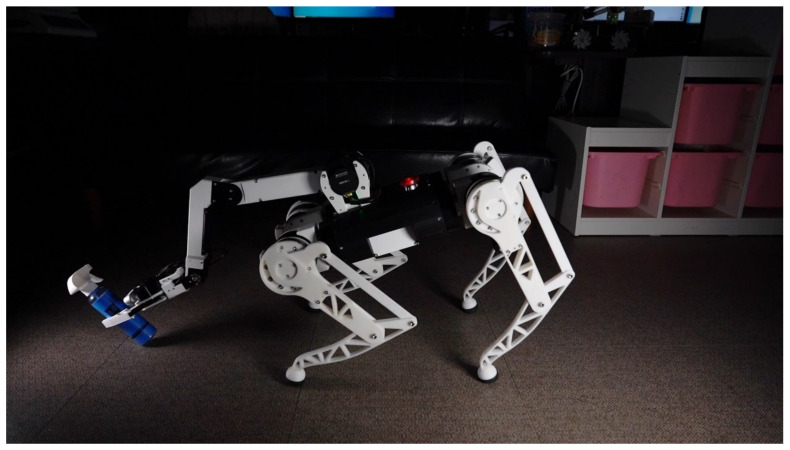
Physical hardware platform for future work.

In our future work, we plan to transition from simulation-based experiments to real-world experimentation employing an actual quadruped robot PADWQ [31], shown in Figure 22, developed at the Pusan National University. Additionally, we will also expand its functionality, such as Natural Language Processing (NLP), pose estimation, human tracking, etc., to encompass a wider array of general-purpose tasks, including HRI. Furthermore, our ongoing efforts will focus on scenarios that involve various terrains, including stairs or thresholds, while concurrently working to reduce task completion times.

## Figures and Tables

**Table 1:** Dimensions of the robot.

Body Length	Body Width	Thigh Length	Calf Length
0.419 m	0.2505 m	0.22 m	0.22 m

**Table 2:** Dimensions of the manipulator.

$d_{1}$	$d_{2}$	$d_{3}$	$d_{4}$	$d_{5}$
0.06 m	0.25 m	0.06 m	0.21 m	0.1 m

**Table 3:** Parameters.

Parameter	Value
$R_{u}$	[0.1 0 0; 0 0.1 0; 0 0 0.1]
$Q_{p}$	[100,000 0 0; 0 150,000 0; 0 0 100,000]
$Q_{\dot{p}}$	[100 0 0; 0 100 0; 0 0 100]
$Q_{R}$	[5000 0 0; 0 5000 0; 0 0 5000]
$Q_{\omega }$	[2 0 0; 0 4 0; 0 0 3]
$N_{hor}$	6
$T_{sw}$	0.3
$T_{st}$	0.1
$T_{pred}$	0.03

Note: $T_{sw}$ , $T_{st}$ , and $T_{pred}$ have the unit [s].

**Table 4:** Results.

Trial	Objects	Success Num	Failure Num	Success Rate	Time
1	Rubik’s cube, clamp, marker, cracker, spoon, Lego, coffee can, baseball, bowl, cup	10	0	100%	19:07
2	tennis ball, bowl, driver, padlock, spoon, sugar, tomato soup, toy gun, mug, brick	8	2	80%	18:55
3	tennis ball, mug, driver, toy gun, padlock, coffee can, Rubik’s cube, tomato soup, bowl, brick	10	0	100%	20:48
4	tennis ball, bowl, padlock, Lego, marker, cracker, brick, mug, tomato soup, Rubik’s cube	10	0	100%	19:59
5	marker, mug, tomato soup, Rubik’s cube, clamp, driver, cup, bowl, coffee can, baseball	10	0	100%	18:10
Average		9.6	0.4	96%	19:24

## Data Availability

Data are unavailable due to privacy.

## Abbreviations

The following abbreviations are used in this manuscript:

DOF	Degree of Freedom
KNN	K-Nearest Neighbor
RANSAC	Random Sample Consensus
HSR	Human Support Robot
HQP	Hierarchical Quadratic Programming
WBC	Whole Body Control
PCA	Principal Component Analysis
COM	Center of Mass
MPC	Model Predictive Control
GRF	Ground Reaction Force
NLP	Natural Language Processing
HRI	Human–Robot Interaction
