# Correction: Yi et al. A Software Platform for Quadruped Robots with Advanced Manipulation Capabilities. *Sensors* 2023, *23*, 8247

**DOI:** 10.3390/s24247968

**Published:** 2024-12-13

**Authors:** Jae-Bong Yi, Shady Nasrat, Min-seong Jo, Seung-Joon Yi

**Affiliations:** Department of Electrical Engineering, Pusan National University, Busan 46241, Republic of Korea; niteofhunter@pusan.ac.kr (J.-B.Y.); shadyloai@pusan.ac.kr (S.N.); jominseong@pusan.ac.kr (M.-s.J.)

## Funding Correction

In the original publication [1], the Funding information was incorrectly printed. The correct Funding information appears below:

**Funding:** This work was supported by a 2-Year Research Grant of Pusan National University.

The authors state that the scientific conclusions are unaffected. This correction was approved by the Academic Editor. The original publication has also been updated.
